# Supplementary material for: Investigating the Influence of Diverse Functionalized Carbon Nanotubes as Conductive Fibers on Paper-Based Sulfur Cathodes in Lithium–Sulfur Batteries
Source: Nanomaterials (Basel). 2024 Mar 7;14(6):484. doi: 10.3390/nano14060484 (PMC10975383; doi:10.3390/nano14060484)
Supplement: Supplementary file 1 [file nanomaterials-14-00484-s001.zip › nanomaterials-2905373-supplementary.pdf]

Supporting Information

Investigating the Influence of Diverse Functionalized Carbon Nanotubes as Conductive Fibers on Paper-Based Sulfur Cathodes in Lithium-Sulfur Batteries

Xuan Ren, Haiwei Wu, Ya Xiao, Haoteng Wu, Huan Wang, Haiwen Li, Yuchen Guo, Peng Xu, Baohong Yang*, Chuanyin Xiong*

Shaanxi Provincial Key Laboratory of Papermaking Technology and Specialty Paper Development, College of Bioresources Chemical and Materials Engineering, Shaanxi University of Science &Technology, Xi'an, China (710021)

Dr. Haiwei Wu, Email: haiweiwufly@163.com, wuhaiwei@sust.edu.cn

Dr. Baohong Yang, Email: yangbaohong@sust.edu.cn

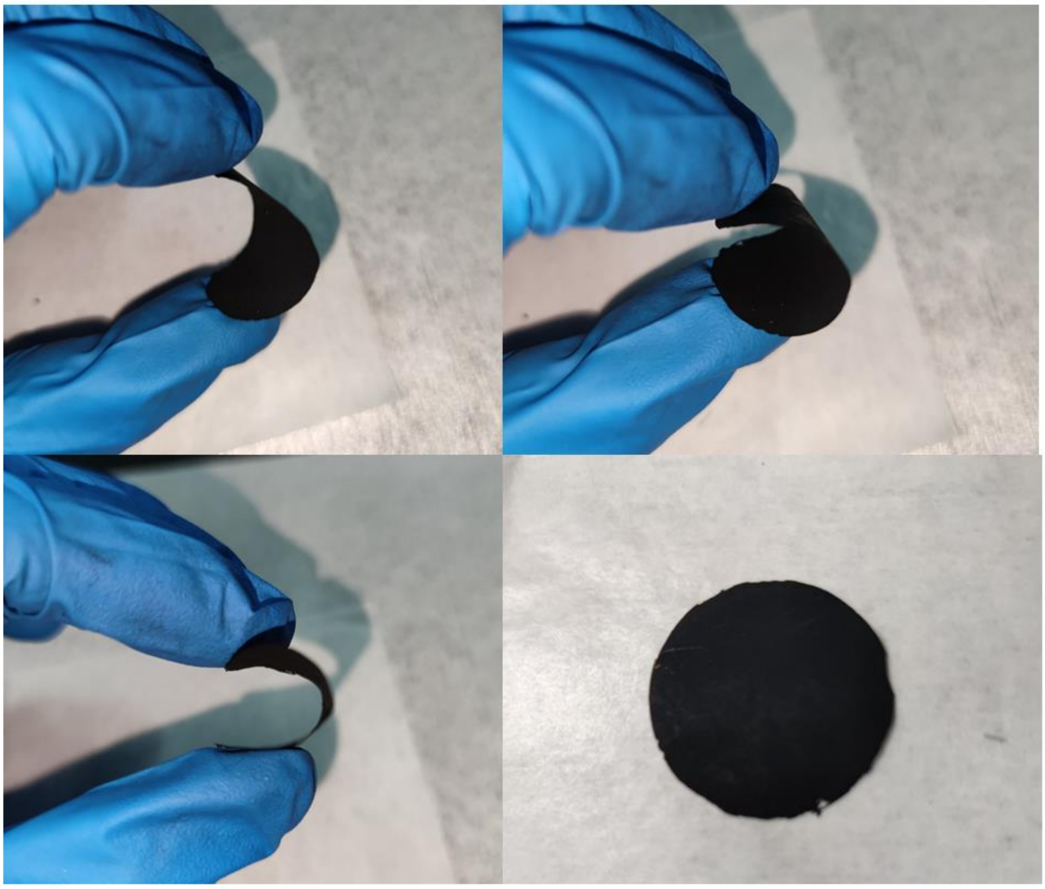

Figure S1 Flexible paper-based sulfur cathode prepared by papermaking process.

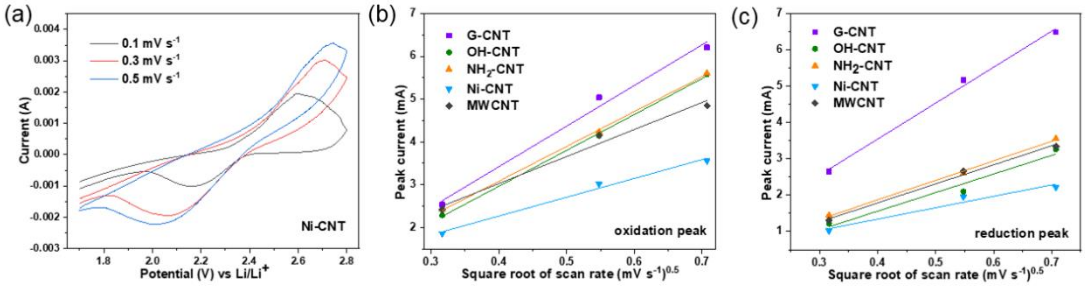

Figure S2 CV curves of Li-S cells assembled by (a) Ni-CNT paper-based electrode at scan rates of 0.1 to 0.5 mV s^{-1}. Fitted curves of peak current versus square root of scan rates for (b) oxidation and (c) reduction peaks.
